# Weak up-regulation of serum response factor in gastric ulcers in patients with co-morbidities is associated with increased risk of recurrent bleeding

**DOI:** 10.1186/1471-230X-11-24

**Published:** 2011-03-16

**Authors:** Hsiu-Chi Cheng, Hsiao-Bai Yang, Wei-Lun Chang, Yi-Chun Yeh, Yu-Ching Tsai, Bor-Shyang Sheu

**Affiliations:** 1 Institute of Clinical Medicine, Medical College, National Cheng Kung University, Sheng Li Road, Tainan, Taiwan; 2 Department of Internal Medicine, Medical College, National Cheng Kung University, Sheng Li Road,Tainan, Taiwan; 3 Department of Pathology, Medical College, National Cheng Kung University, Sheng Li Road, Tainan, Taiwan; 4 Department of Pathology, Ton-Yen General Hospital, Xian Zheng 2ndRoad, Zhubei, Hsinchu, Taiwan; 5 Institute of Basic Medical Sciences, Medical College, National Cheng Kung University, Sheng Li Road, Tainan, Taiwan

## Abstract

**Background:**

Serum response factor (SRF) is crucial for gastric ulcer healing process. The study determined if gastric ulcer tissues up-regulate SRF and if such up-regulation correlated with co-morbidities and the risk of recurrent bleeding.

**Methods:**

Ulcer and non-ulcer tissues were obtained from 142 patients with active gastric ulcers for SRF expression assessed by immunohistochemistry. Based on the degree of SRF expression between these two tissue types, SRF up-regulation was classified as strong, intermediate, and weak patterns. The patients were followed-up to determine if SRF up-regulation correlated to recurrent bleeding.

**Results:**

Gastric ulcer tissues had higher SRF expression than non-ulcer tissues (*p *< 0.05). Patients with strong SRF up-regulation had lower rates of stigmata of recent hemorrhage (SRH) on the ulcer base than the others (*p *< 0.05). Multivariate logistic regression confirmed that co-morbidities and weak SRF up-regulation were two independent factors of recurrent gastric ulcer bleeding (*p *< 0.05). Combining both factors, there was an 8.29-fold (95% CI, 1.31~52.62; *p *= 0.03) higher risk of recurrent gastric ulcer bleeding.

**Conclusions:**

SRF expression is higher in gastric ulcer tissues than in non-ulcer tissues. Weak SRF up-regulation, combined with the presence of co-morbidities, increase the risk of the recurrent gastric ulcer bleeding.

## Background

Peptic ulcer bleeding is a common and potentially lethal disease with mortality rates as high as 8-10%. The high mortality is accounted for by senility, etiology, severity of bleeding, co-morbidities, and the presence of stigmata of recent hemorrhage (SRH) [1]. SRH is a classical clinical assessment that predicts the development of recurrent peptic ulcer bleeding if the fading time of SRH is 4.1 ± 2.1 days after first bleed [2,3]. However, patients with co-morbid illnesses are prone not only to having poor ulcer healing but also to delayed recurrent bleeding [4-7]. For patients at risk, it is necessary to validate the possible molecule mechanisms related to ulcer healing.

Serum Response Factor (SRF), a transcription factor protein, binds to serum response elements to control particular genes expressions for peptic ulcer healing [8-11]. The healing process of gastric ulcers is complex, requiring the restoration and proliferation of epithelial cells, fibroblasts, macrophages, and endothelial cells [12-15]. SRF reportedly promotes gastric ulcer healing by stimulating the proliferation and differentiation of such cells [15,16]. Therefore, it is important to determine the clinical significance of SRF in gastric ulcers healing, especially in patients with SRH and co-morbidities.

This study aimed to determine whether SRF expression in gastric ulcer tissues is higher than that of antral non-ulcer tissues, and if SRF expression in gastric ulcer tissues is related to *Helicobacter pylori *(*H. pylori*) infection, non-steroidal anti-inflammatory drugs (NSAID) intake, or others. This study is the first to validate the correlation of SRF up-regulation with the presence of SRH and determine if it is independently predictive of the recurrent gastric ulcer bleeding.

## Methods

### Patients and study design

Patients with or without co-morbidities who received upper gastroscopy for melena, hematochezia, or hematemesis and being disclosed gastric ulcers were consecutively enrolled. We enrolled patients with gastric ulcers but not duodenal ulcers because gastric ulcers biopsy is performed according to routine practice at the investigational center. The co-morbidities included congestive heart failure, coronary artery disease, disseminated malignancy, liver cirrhosis, acute renal failure, chronic kidney disease, end-stage renal disease requiring hemodialysis, chronic obstructive pulmonary disease, pneumonia, restrictive lung disease, sepsis, and new onset cerebro-vascular accident.

Patients were excluded if they had tumor bleeding or ulcer bleeding due to mechanical factors (i.e. induction of gastrostomy tube), use of anticoagulant, or failure to establish hemostasis during upper gastroscopy.

The SRH was defined by Forrest et al. [2]. Patients with bleeding gastric ulcers and classified as Forrest classification Ia (arterial bleeding), Ib (oozing bleeding), IIa (non-bleeding visible vessels), IIb (adherent blood clots), to IIc (hematin) of gastric ulcers bleeding were treated by standardized endoscopic injection therapy with diluted epinephrine 1:10000 or normal saline regardless of combined therapy with heater probe, argon plasma coagulation, band ligation, or hemoclip therapy to eradicate the vessel. After hemostasis by endoscopic therapy, patients received an 80 mg bolus injection of intravenous omeprazole (LOSEC, AstraZeneca AB, Södertälje, Sweden), then intravenous omeprazole infusion 8 mg/h for 3 day. After omeprazole infusion, oral esomeprazole (NEXIUM, AstraZeneca AB) 40 mg daily was given until the end of follow-up.

Gastric mucosal biopsies were performed under direct vision with the Olympus gastroscopy (Olympus GIF-XQ 260 endoscope, Olympus Optical Co. Ltd, Tokyo, Japan) within 12 h after bleeding. Patients with Forrest classification Ia to IIb of gastric ulcers bleeding underwent second look gastroscopy for gastric mucosal biopsy via standard biopsy forceps (Olympus FB-25K-1) for histology studies three days later. Eight biopsies were taken: six from the ulcer edge as ulcer tissues and two from the antral mucosa that was macroscopically normal, about 2 cm from the pylori ring and at least 2 cm from the ulcer, as antral non-ulcer tissues [17,18].

The patients were diagnosed with *H. pylori *infection either by positive rapid urease test (CLO test, Kimberly-Clark, Draper, Utah, USA) or histology as before [17-19]. NSAID intake was defined as continuous or sporadic intake more than 3 times a week during the past 4 weeks. The ethics committee of National Cheng Kung University, Taiwan, approved the study design and participants provided informed consent. The institutional review board is "Pathogenic roles and clinical relevance of TGF-β1 and SRF in peptic ulcer patients with comorbid illnesses".

### Outcome measures

All of the patients were followed-up as possible until 28 days after the first episode of melena, hematochezia, or hematemesis. The primary end-point was recurrent bleeding within this period. Documented recurrent bleeding should fulfill one of the following conditions: (i) continuous melena, hematochezia, or the presence of recurrent bloody aspirates through the nasogastric tube; and (ii) relapse of hemodynamic instability, including systolic blood pressure <90 mmHg, heart rate >120 beats/min, or hemoglobin drop >2 g/dL. We did not perform routine second look endoscopy in the absence of above clinical signs of re-bleeding to avoid misclassification of non-significant re-bleeding [20].

### Immunohistochemistry Studies for Gastric SRF Expression

Tissue immune-histochemical staining was performed using monoclonal antibody of SRF (Santa Cruz Biotechnology, Inc. Santa Cruz, CA, USA). The gastric tissue was fixed in 10% buffered formalin. The specimen was embedded in paraffin, serially sectioned at 4 μm thickness, placed onto the microscope slide, and deparaffinized in xylene and dehydrated in a graded series of ethanol. The specimen was immersed for 20 minutes in 3% hydrogen peroxide to stop endogenous peroxidase activity and antigen retrieval with DakoCytomation Target Retrieval Solution (Dako, Carpinteria, CA, USA). The non-specific binding site was saturated with 2.5% bovine serum albumin. The tissue section was treated with primary antibody against SRF at a dilution of 1:400 and then incubated overnight in a humidified chamber at 4°C.

The SuperPicTure™Polymer Detection Kit (Invitrogen, Carlsbad, CA, USA) was adapted for blocking, linkage, and labeling for staining according to the manufacturer's instructions. 3-amino-9-ethylcarbazole was used as the chromogen. The section was then counter-stained with hematoxylin. The colon ulcer tissue was used for positive control.

**Figure 1:**
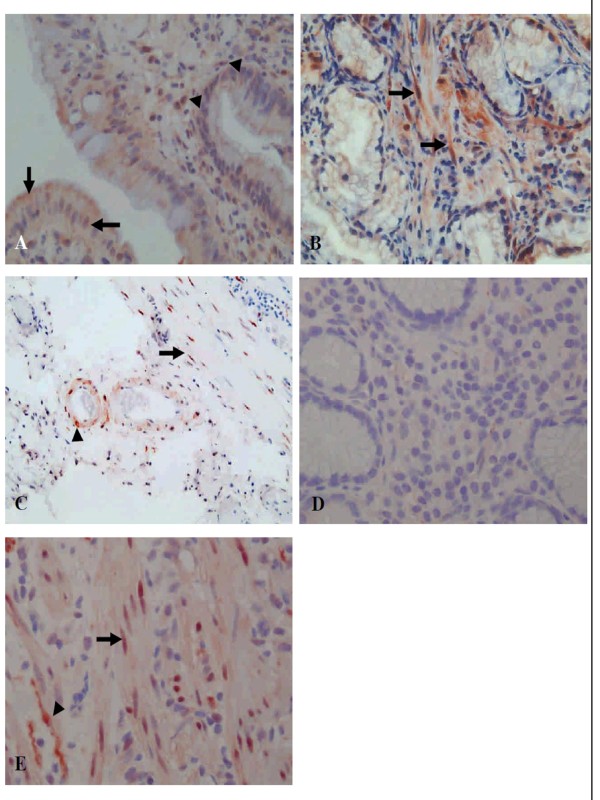
**Representative immune-histochemical staining of SRF expression**. **(A) **High nuclear and cytoplasmic SRF intensity of regenerative epithelial cells (arrow), some mononuclear inflammatory cells and myofibroblasts (arrowhead) of the lamina propria in gastric ulcer tissues (Magnification × 2400). **(B) **Increased nuclear and cytoplasmic SRF intensity of smooth muscle cells (arrow) between the deep glands (× 1200). **(C) **Nuclear staining of SRF in smooth muscle cells of the muscularis mucosa (arrow) and vascular wall (arrowhead) (× 600). **(D) **Low SRF intensity in non-ulcer tissues (× 2400). **(E) **The positive control of colon ulcer tissues indicates positive nuclear staining in smooth muscle cells (arrow) and nerve (arrowhead).

The same pathologist blinded to the patients' clinical background scored the immune-histochemical staining of SRF. The expression grades of SRF staining were scored semi-quantitatively according to the percentage of superficial epithelial cells or mononuclear cells of the lamina propria positively stained. The score ranged from 0-4 in intensity, listed as 0 (negative), 1 (< 5% cells), 2 (5-29% cells), 3 (30-59% cells), or 4 (≥60% cells) [21,22]. Mucosal smooth muscle cells were graded as 1 (baseline staining in capillary muscle), 2 (increase of inter-glandular muscle), and 3 (increase with nuclear staining) (Figure 1). A score ≥2 was defined as high SRF expression with >5% cells of the superficial epithelium and mononuclear cells of the lamina propria positively stained, or with positively stained inter-glandular smooth muscle cells. A score <2 was defined as low SRF expression.

The net increase of SRF intensity between ulcer tissues and non-ulcer tissues within the same individual was also compared. The net increase of SRF intensity was calculated as SRF intensity of the ulcer tissue minus that of the non-ulcer tissue in the superficial epithelium, mononuclear cells of the lamina propria, and mucosal smooth muscle cells, respectively. Based on the degree of net increase of SRF intensity, SRF up-regulation on ulcer tissues was classified into strong, intermediate, and weak patterns. Strong up-regulation of SRF on ulcer tissues was defined as a uniform net-positive increase of SRF intensity over the gastric epithelium, mononuclear cells and muscle cells. In contrast, weak SRF up-regulation on ulcer tissues was defined by the presence of two net-negative increases of SRF intensity compared to non-ulcer tissues. The other patterns of SRF net changes were accordingly defined as intermediate up-regulation.

### Statistical analysis

The Student *t *test, Fisher's exact test, one-way ANOVA and Pearson χ^2 ^test with McNemar's correction were used as appropriate. The Mann-Whitney *U *test was applied to assess SRF intensity in the different study groups. All of the tests were two-tailed, with *p *< 0.05 taken as significant.

## Results

### Demographic background of the study patients

From August 2003 to December 2007, 142 patients (58 women and 84 men) with mean age of 66 years and gastric ulcers in the active stage were prospectively consecutively enrolled. Of the 142, 132 (93.0%) patients' ulcers located on the antrum, others located on the body. Seventy-eight (54.9%) patients had Forrest classification Ia to IIc SRH, and 94.2% (49/52) of Ia to IIb SRH were treated with endoscopic hemostatic monotherapy or dual therapy. The etiologies of gastric ulcers included 45 *H. pylori*-infected ulcers, 30 NSAID-related ulcers, 46 *H. pylori*-infected and NSAID-related ulcers, and 21 other causes induced ulcers.

### Gastric ulcer tissues had higher SRF expression than non-ulcer tissues

SRF was predominantly stained in nuclei or cytoplasm of regenerative superficial epithelium and mononuclear inflammatory cells and myofibroblasts of the lamina propria (Figure 1A and 1B). SRF was also stained in nuclei of smooth muscle cells of the muscularis mucosa and vascular wall (Figure 1C). The rates of high SRF expression were significantly higher in ulcer tissues (Figure 1A) than in non-ulcer tissues (Figure 1D), including the superficial epithelium (54.2% *vs*. 30.3%, *p *< 0.01), mononuclear cells of the lamina propria (69% *vs*. 58.5%, *p *= 0.021), and mucosal smooth muscle cells (80% *vs*. 68.1%, *p *= 0.024) (Table 1). Moreover, for both ulcer and non-ulcer tissues, the rates of high SRF intensity on mucosal smooth muscle cells or mononuclear cells of the lamina propria were higher than those on the superficial epithelium (*p *< 0.01).

**Table 1:** Gastric ulcer tissues had higher SRF intensity than non-ulcer tissues

Gastric histology (n = 142)	High SRF intensity (n, %)		*P *value^†^
		
	Ulcer	Non-ulcer	
Superficial epithelium	77 (54.2%)	43 (30.3%)	<0.01
Mononuclear cells of the lamina propria	98 (69.0%)	83 (58.5%)	0.021
Mucosal smooth muscle cells	112 (80.0%)	96 (68.1%)	0.024
*P *value	<0.01^‡, §^, 0.035¶	<0.01^‡, §^, 0.067¶	

^†^*P *values indicated the significant difference of the rates of the high SRF intensity on ulcer tissues as compared with antral non-ulcer tissues (by McNemar's test). ^‡^Superficial epithelium *vs*. mononuclear cells of the lamina propria. ^§^Superficial epithelium *vs*. mucosal smooth muscle cells. ¶Mononuclear cells of the lamina propria *vs*. mucosal smooth muscle cells. SRF: serum response factor.

### Factors related to up-regulation of SRF intensity on gastric ulcer

Based on the degree of the net increase of SRF intensity, SRF up-regulation were classified into strong (n = 18), intermediate (n = 83), and weak (n = 41) patterns. The most impressive finding was that patients with strong up-regulation of SRF intensity had lower rate of Forrest classification Ia to IIc SRH than patients with either intermediate or weak SRF up-regulation (*p *< 0.05) and had a trend of less co-morbid illness (*p *= 0.14). Nonetheless, there were no differences in age, ulcer size, endoscopic hemostatic therapy, mean hemoglobin, platelet counts, percentage of hypo-albuminemia <3 g/dL, and serum creatinine ≥1.5 mg/dL among these patients with different degrees of SRF up-regulation (*p *> 0.05, Table 2). However, SRF intensities were similar between patients with serum albumin <3.0 and those with ≥3.0 g/dL (*p *> 0.05).

**Table 2:** Demographic and clinical parameters correlated with different degrees of SRF up-regulation on gastric ulcers

Up-regulation of SRF	Strong (n = 18)	Intermediate (n = 83)	Weak (n = 41)	*P* value^†^
Female: Male	7 : 11	29 : 54	22 : 19	0.13
Mean age (yr)	62.3	66.4	67.0	0.53
Ulcer characteristics (n)				
SRH, Forrest classification Ia to IIc (%)	22.2	61.4	58.5	0.01
Mean ulcer size (cm)	1.51	1.47	1.70	0.65
Endoscopic hemostatic therapy in patients with Forrest classification Ia to IIb SRH (%)	100	91.9	100	0.52
*H. pylori *infection (%)	61.1	62.7	68.3	0.80
NSAID user (%)	55.6	50.6	58.5	0.70
Comorbidity (%)	33.3	57.8	58.5	0.14
Mean Hb (g/dL)^‡^	10.2	10.0	9.6	0.71
Platelet (mm^3^)^‡^	300.4	244.7	247.3	0.24
Serum albumin <3 g/dL (%)^‡^	37.5	26.4	36.0	0.62
Serum Creatinine ≥ 1.5 mg/dL (%)^‡^	20.0	26.3	33.3	0.58

Up-regulation of SRF indicated the distribution of net-positive increase of SRF intensity in ulcer tissues than in non-ulcer tissues of superficial epithelium, mononuclear cells of the lamina propria, and mucosal smooth muscle cells, respectively. SRF: serum response factor; SRH: stigmata of recent hemorrhage; *H. pylori*: *Helicobacter pylori*; NSAID: non-steroidal anti-inflammatory drugs; Hb: hemoglobin. ^†^One-way ANOVA and Pearson χ^2 ^test were used as appropriate. ^‡^Reference range: Hb level, 13.5-17 g/dL; platelet count; 138.1-353.4 × 10^3^/cmm; serum albumin level, 3.5-5 g/dL; serum creatinine, 0.7-1.5 mg/dL.

**Figure 2:**
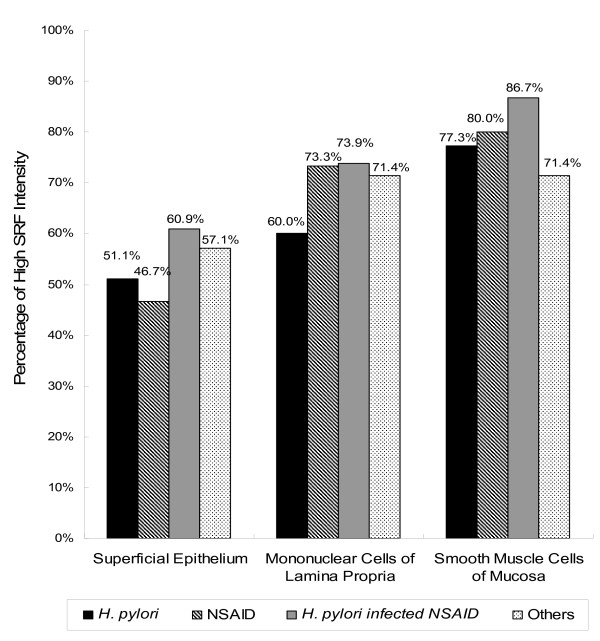
**Percentages of high SRF intensity of gastric ulcers among patients with different etiologies**. The rates of high SRF intensity of gastric ulcers were similar among patients with *H. pylori*-infected ulcers, NSAID-related ulcers, *H. pylori*-infected and NSAID-related ulcers, and others, either in the superficial epithelium, mononuclear cells of the lamina propria, and mucosal smooth muscle cells (*p *> 0.05). SRF, serum response factor; *H. pylori*, *Helicobacter pylori*; NSAID, non-steroidal anti-inflammatory drugs.

There were no differences in *H. pylori *infection or NSAID use among patients with different degrees of SRF up-regulations (*p *> 0.05) (Table 2). The rates of high SRF intensity of gastric ulcers were similar among patients with *H. pylori*-infected ulcers, NSAID-related ulcers, or *H. pylori*-infected and NSAID-related ulcers (*p *> 0.05) (Figure 2).

### Weak SRF up-regulation independently related with recurrent gastric ulcer bleeding

**Figure 3:**
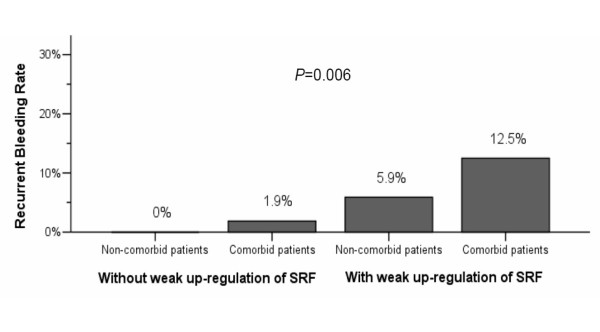
**Recurrent bleeding rates of gastric ulcers were increased with weak SRF up-regulation and co-morbidities**. SRF, serum response factor.

Underlying medical co-morbidity and the presence of weak SRF up-regulation on ulcer tissues were not only significant univariate factors (Table 3, *p *< 0.05) but also independent risk factors of recurrent bleeding (*p *< 0.05). Age ≥60 years, *H. pylori *infection, or NSAID use were not risk factors of recurrent bleeding (*p *> 0.05). The recurrent bleeding rates of gastric ulcers increased stepwise from 0% in non-co-morbid patients without weak SRF up-regulation, 1.9% in co-morbid patients without weak SRF up-regulation to 5.9% in non-co-morbid patients with weak SRF up-regulation and up to 12.5% in co-morbid patients with weak SRF up-regulation (*p *= 0.006) (Figure 3). Combining both factors, the risk of recurrent gastric ulcer bleeding in co-morbid patients with weak SRF up-regulation increased by 8.29-fold (95% CI, 1.31~52.62; *p *= 0.03).

**Table 3:** The significant univariate analysis and multivariate logistic regression to determine factors associated with recurrent bleeding

Related factors *Univariate analysis*	Recurrent bleeding rates (%)	Odds ratio (95% CI)	*P* value^†^
SRH of Forrest classification Ia to IIc *vs*. none	6.4 *vs*. 0	-	0.06
Comorbidities *vs*. none	14.3 *vs*. 1.7	9.92 (1.55 ~ 63.49)	0.02
Weak up-regulation of SRF *vs*. others	9.8 *vs*. 1.0	10.81 (1.17 ~ 99.89)	0.02

***Multivariate logistic regression***	Coefficient (SE)	95% CI	*P *value^‡^

Comorbidities *vs*. none	2.29 (1.05)	1.25 ~ 77.71	0.03
Weak up-regulation of SRF *vs*. others	2.36 (1.20)	1.02 ~ 111.07	0.048

SRH: stigmata of recent hemorrhage; SRF, serum response factor; CI, Confidence interval; SE, standard error. ^†^*P *value was assessed by two-tailed Fisher's exact test. ^‡^Indicated the significance of the multivariate logistic regression.

The rates of weak SRF up-regulation on gastric ulcers increased in a trend of patients with neither recurrent bleeding nor co-morbidity, without recurrent bleeding but with co-morbidity, with recurrent bleeding but without co-morbidity, and with both recurrent bleeding and co-morbidity (25.4%, 28.4%, 75%, and 100%, respectively; *p *= 0.03).

## Discussion

Emerging evidences suggest that the incidences of idiopathic peptic ulcer are increasing in Eastern and Western countries, at 18.8% and 44%, respectively [4,23-25]. Most of these patients (≥70%) have co-morbid illnesses and half are severe or life-threatening systemic disorders, such as major organ failure and malignancy [4,23]. These patients have higher risks of ulcer recurrence and bleeding in one-month, 12-month, or 7-year follow-up [4,5,7,26]. Although the presence of SRH is a good measure for predicting recurrent bleeding, it cannot predict delayed recurrent bleeding in such patients at risk. It is thus worthy to test whether there is any host background factor other than SRH that can act as a determinant of recurrent bleeding.

Gastric ulcer healing should be assisted with granulation tissues developing at the ulcer base, which consist of proliferating fibroblasts and endothelial cells that restore the lamina propria [12-14,27]. Such healing processes are related to the expression of immediate early genes (eg, *c-fos*) and muscle-specific genes (eg, *smooth muscle α-actin, smoothelin*), containing serum response element (SRE) to be regulated by SRF [28-31]. By comparing between ulcer tissues and non-ulcer tissues within the same individual, this study is highly original in revealing how SRF intensity can be commonly up-regulated in ulcer tissues than in non-ulcer tissues. Such finding implies that SRF has a potential role in gastric ulcer healing. However, because of varying degrees of SRF up-regulation, there is a need to determine its real clinical significance.

Patients with weak SRF up-regulation in ulcer tissues have significantly higher rates of Forrest classification Ia to IIc SRH in gastric ulcers (*p *< 0.01) and this trend becomes more common with co-morbid illnesses (*p *= 0.14) (Table 2). Since SRH and co-morbidities carry higher risks of recurrent bleeding in previous studies [1,6,19,26,32,33], it will be interesting to determine if weak SRF up-regulation on ulcer tissues is associated with recurrent gastric ulcers bleeding.

Aside from serving as significant univariate factors, both the weak up-regulation of SRF and the presence of co-morbidity can be independent risk factors for predicting recurrent gastric ulcer bleeding (*p *< 0.05) (Table 3). As both factors can independently determine recurrent bleeding of gastric ulcers (Table 3), it is not surprising to show that their combined effect increases the risk of recurrent gastric ulcer bleeding (Figure 3).

SRF has potential healing benefits in gastric ulcers, and our result showed weak SRF up-regulation predisposes to increased risk of recurrent gastric ulcer bleeding. SRF makes progress in healing processes such as promotes migration and proliferation of gastric epithelial cells, smooth muscle cells, and endothelial cells [15,16]. SRF deficiency inhibits vascular endothelial growth factor (VEGF)-stimulated endothelial cell migration and proliferation and inhibits angiogenesis [34]. Further study are promising to test whether weak SRF up-regulation have a decreased VEGF expression and poor angiogenesis. Moreover, it is promising to conduct further study to validate the correlation between the proliferation index such as proliferating cell nuclear antigen (PCNA) or Ki-67 and the SRF expression at the ulcer edge.

SRF is activated by extracellular stimulations such as serum and mitogens through a ternary complex factors-dependent pathway involving the ras-raf-mitogen-activated protein kinase (MAPK)-extracellular signal-regulated kinase (ERK) cascade and other pathways [35]. *H. pylori *infection activates the SRE-driven *c-fos *transcription in epithelial cells through the activation of ERK/MAPK cascade [36,37]. It is well known that NSAID suppresses the ERK signaling pathway [38], however, little is known about the effect of NSAID on SRE-driven genes transcription. Nevertheless, our study showed SRF expression on gastric ulcers is not different between patients with *H. pylori *infection and NSAID users (Figure 2). The possible reasons may be whatever the etiology of gastric ulcers is, SRF is triggered and activated by wounding. As *H. pylori *infection and NSAID use are the two major leading etiologies of gastric ulcers, assessing the up-regulation of SRF can be widely applied for most patients.

Although co-morbidity remains an important risk factor of recurrent bleeding of gastric ulcers [1,6,7], the long-term efficacy of oral proton pump inhibitors (PPI) or other gastro-protective agents such as misoprostol to prevent ulcer recurrence and bleeding is uncertain. PPI had been proven to promote gastric epithelial cell proliferation and migration, and can inhibit pro-inflammatory response, which is pH-independent [39-41]. Therefore, the SRF role may be as a possible molecule mechanism and potential therapeutic target for high-risk patients.

Moreover, the other important issue is who will benefit from such therapy. The risk of recurrent bleeding is rather low for those without weak SRF up-regulation. In contrast, the risk increases by 8.29-fold for those with both co-morbid illnesses and weak up-regulation of SRF (Figure 3). Data here corroborates the assessment of SRF expression in gastric ulcer tissues as meaningful and important in identifying a subset of patients who carry higher risk of recurrent bleeding. For such patients, more aggressive bleeding control such as applying long term oral PPI or improving the weak up-regulation of SRF on gastric ulcers may be mandatory.

Most gastric ulcer bleeding is self-limited, however, a subset of patients have recurrent bleeding. The prognosis factors are old age (>60 y/o), comorbidity, large ulcers (>1.0 cm), SRH, and others [1,2,42,43]. Endoscopic hemostatic therapy prevents recurrent bleeding and mortality [44]. In order to study the correlation of SRF expression and co-morbidity, we enrolled patients who had the similar characteristics of age (70% of patients ≥ 60 y/o) and ulcer size (62% of patients had ulcers ≥ 1.0 cm) and almost patients (94.2%) with Forrest classification Ia to IIb SRH underwent standardized primary endoscopic hemostatic therapy. It may be the reasons why our data show lack of correlation between SRF expression and age, ulcer size, and endoscopic hemostatic therapy.

## Conclusions

In summary, gastric ulcer tissues exert different degrees of SRF up-regulation. The weak up-regulation of SRF on ulcer tissues and the presence of co-morbidities independently increase the risk of recurrent gastric ulcer bleeding. Especially in patients with co-morbidities, improving the up-regulation of SRF may be a potential therapeutic target for preventing recurrent bleeding.

## List of abbreviations used

SRH: Stigmata of recent hemorrhage; SRF: Serum response factor; *H. pylori*: *Helicobacter pylori*; NSAID: non-steroidal anti-inflammatory drugs; SRE: Serum response element; VEGF: Vascular endothelial growth factor; PCNA: Proliferating cell nuclear antigen; MAPK: Mitogen-activated protein kinase; ERK: Extracellular signal-regulated kinase; PPI: Proton pump inhibitor

## Competing interests

The authors declare that they have no competing interests.

## Authors' contributions

**Guarantor of the article**: BSS

**Specific author contributions**: HCC and BSS initiated the study and coordinated the conduct of the whole study. HCC wrote and BSS refined the manuscript. HBY reviewed the pathology and SRF expression. HCC and WLC enrolled and followed-up the patients in clinics. YCT and YCY conducted the immunohistochemistry. All authors read and approved the final manuscript.

## Authors' information

Hsiu-Chi Cheng, MD, PhD: Institute of Clinical Medicine, Department of Internal Medicine, Medical College, National Cheng Kung University, Tainan, Taiwan.

Hsiao-Bai Yang, MD: Department of Pathology, Medical College, National Cheng Kung University, Tainan; Department of Pathology, Ton-Yen General Hospital, Hsinchu, Taiwan.

Wei-Lun Chang, MD: Institute of Clinical Medicine, Department of Internal Medicine, Medical College, National Cheng Kung University, Tainan, Taiwan.

Yi-Chun Yeh, MS: Institute of Clinical Medicine, Institute of Basic Medical Sciences, Medical College, National Cheng Kung University, Tainan, Taiwan.

Yu-Ching Tsai, MD: Institute of Clinical Medicine, Medical College, National Cheng Kung University, Tainan, Taiwan.

Bor-Shyang Sheu, MD: Institute of Clinical Medicine, Department of Internal Medicine, Medical College, National Cheng Kung University, Tainan, Taiwan.

## Pre-publication history

The pre-publication history for this paper can be accessed here:

http://www.biomedcentral.com/1471-230X/11/24/prepub
